# The Writing's on the Wall: On Health Inequalities, Migrants, and Coronavirus

**DOI:** 10.3389/fpubh.2020.00505

**Published:** 2020-09-11

**Authors:** Ahmed Nabil Shaaban, Barbara Peleteiro, Maria Rosario O. Martins

**Affiliations:** ^1^Global Health and Tropical Medicine (GHTM), Institute of Hygiene and Tropical Medicine (IHMT), NOVA University of Lisbon, Lisbon, Portugal; ^2^EPIUnit—Instituto de Saúde Pública, Universidade Do Porto, Porto, Portugal; ^3^Departamento de Ciências da Saúde Pública e Forenses e Educação Médica, Faculdade de Medicina da Universidade Do Porto, Porto, Portugal

**Keywords:** migrants, ethnic minorities, health inequalities, COVID-19, Europe

Migrants and ethnic minorities who lack access to health services and information are particularly vulnerable to the impact of COVID-19. In this opinion, we aimed to explain how these challenges can affect the spread of COVID-19 in the European Union and the United Kingdom, and to stress the importance of universal and equitable access to healthcare services during and after the COVID-19 crisis.

On March 13, 2020, the World Health Organization declared Europe as the new epicenter of the COVID-19 pandemic (1). However, the disease has exposed existing health inequalities in specific communities, which may experience a substantial burden of the disease, namely migrants and ethnic minorities. In the United States (US) and Europe, hospitalization and mortality rates among ethnic minorities have been markedly greater than that observed in each country's host population. In the US, the hospitalization rate related to COVID-19 among the African American community, which accounts for only 13% of the US population, was approximately five times higher than that of the non-Hispanic white population, while the mortality rate among the same community was more than twice the non-Hispanic white population (2, 3). Likewise, the Hispanic minority population, which accounts for only 18% of the US population, had an hospitalization rate four times higher than that observed in the non-Hispanic white population (2). Ethnic minorities in the US are well known to be subjected to social and health inequalities, which have resulted in adverse health outcomes and morbidities, especially when compared to the White ethnic majority (4–6). Similar findings have been observed in both the United Kingdom (UK) and Norway. In the latter, migrants, who represent only 15% of the total population, account for 25% of the total confirmed cases of the virus (7). In the UK, migrants and ethnic minorities, who represent only 17% of the UK population (8), account for more than 30% of admissions to intensive care units (9) and for more than 50% of COVID-19 hospitalizations among pregnant women in particular (10). Moreover, the mortality rate among Black males in the UK was more than three times higher than the mortality among their White counterparts, while the mortality rate among Black females was more than twice higher (11).

These findings may be worrying for the European Union (EU) countries and the UK, given that ~30 million migrants currently live in these countries (12, 13). Unlike the UK and the US, most of the EU countries do not collect health data based on ethnicity (14, 15), limiting the information on the health of migrants in these countries (16). However, it is well-known that the health systems in these countries are already plagued with inequalities that have a disproportionate effect on migrants. These inequalities include unequal access to services, and poorer health outcomes when compared to host communities; in particular, migrants report higher incidences of morbidity and mortality for a range of chronic diseases (17–22). Migrants may encounter several barriers to access healthcare services in Europe, which may be related to the patient, the provider and the healthcare systems (23, 24). Key barriers reported by migrant populations include financial and administrative barriers, language limitations, cultural differences, and lack of knowledge regarding how the healthcare system operates in the new country of residence (23–25). Moreover, a particular migrant group who may find themselves disproportionately affected by the current COVID-19 pandemic in Europe are undocumented migrants. Unlike documented migrants, undocumented migrants are less likely to be entitled to healthcare services due to national regulations limiting their access (26–29). Moreover, they have a higher probability of suffering from poor health outcomes and worse neglected health conditions due to fear of being deported if they are discovered by authorities (30, 31). This is of particular concern due to the evidence indicating that this migrant group is less likely to access critical preventive services, and hence, there is a higher potential for public health problems among them specially with regards to infectious diseases (27). Moreover, the lack of data on health based on ethnicity in most EU countries limits the possibility of conducting studies on inequalities among these vulnerable populations, hence, halts further improvements of migrants' health (15).

Lessons from previous epidemics and pandemics show that pre-existing health inequalities and underlying health conditions could disproportionally affect morbidity and mortality among disadvantaged populations (32). There is also strong evidence that migrants and ethnic minorities specifically experience a substantial burden when it comes to infectious diseases owing to the lack of access to preventive healthcare services and information (33). As a result, migrants could be particularly vulnerable to the impact of COVID-19.

Moreover, pre-existing social and socioeconomic inequalities among migrants in Europe, such as housing, income, poverty, employment, and access to social security, can also contribute to the high rates of COVID-19 infections among migrants. Historically, social determinants of health and socioeconomic factors play an important role among socially disadvantaged populations during epidemics as it predisposes high risk populations to unfavorable health outcomes (32, 34). Examples of these socioeconomic disparities among migrants in Europe should raise several alarms. The unemployment rate among migrants living in the European Union (EU) is 12.3% compared to only 6.0% among the native-born population (35). Of particular importance here is that some EU member states, which were among the most affected by COVID-19, also have among the highest recorded rates of unemployment among migrants, such as Spain (19.9%), Sweden (17.3%), France (14.7%), and Belgium (13.8%) (36). With regards to poverty, migrants in the EU are more likely to experience poverty and social exclusion than other foreign EU citizens and native-born populations, 45, 29, and 21%, respectively (37). Additionally, the risk of monetary poverty is highly concentrated among non-EU citizens when compared to native-born populations, 38 and 15%, respectively. Non-EU citizens also exhibit a high incidence of severe material deprivation when compared to nationals, 13 and 6%, respectively (20).

Housing, which is another key factor for living conditions and well-being, represents another aspect of disparity among migrants in Europe. For example, non-EU citizens are more likely to live in overcrowded places when compared to nationals, 35 and 17%, respectively (38). Moreover, migrants in the EU report a higher rate of housing cost overburden (24%) as compared to native populations (9%) (21). These economic constraints among migrants and the lack of knowledge regarding the rental market, which can be imposed by language or social barriers, may affect the choice of acquiring adequate housing (39). It is important to highlight that some ethnic minorities in Europe, who were disproportionately affected by COVID-19, are known to live in overcrowded households. For example, the Somali community in three Nordic countries, namely Norway, Sweden, and Finland, is known to live in overcrowded households that are highly overrepresented in confirmed positive COVID-19 cases, with estimates being 10 times higher than the observed in the general population in Norway and Helsinki (Finland), and seven times higher than the general population in Sweden, making them the largest migrant community testing positive for COVID-19 in these countries (28). Another study from the UK found that Black, Asian and Minority Ethnic (BAME), who are more likely to live in overcrowded households (40), are disproportionately affected by COVID-19 (41). These aggregates of socioeconomic inequalities and the pre-existing health disparities among migrants, will not only increase their vulnerability to infection and disproportionally affected by the disease, but will also limit the governments' capability of applying the required public health measures to contain the spread of infection by COVID-19.

Another aspect that requires great precaution is how any economic downturn can affect the migrant labor forces in Europe. The expectations of an inevitable global recession due to COVID-19 require a careful understanding of how any potential financial crisis may affect the health of international labor workers in the EU and the UK. It is estimated that almost 40 million people are migrant workers in an area mainly formed by the EU-27 countries and the UK, which accounts for 23.9% of the world's international workforce (42). We might expect higher rates of unemployment, job loss and poverty among migrants as a result of any potential economic recession due to COVID-19. Moreover, previous studies have shown that migrants are among the most affected by infectious diseases and epidemics during economic crises due to worsening living conditions, and lack of access to healthcare and treatment (43). Ensuring that any potential economic recession will not result in budget cuts that may affect the health sector for migrants is crucial. For example, in the face of the economic crisis that began in 2008 and the subsequent financial recession in the following years, several European countries adopted drastic measures that led to restrictions in access to healthcare and sometimes the exclusion of migrants from health plans, especially undocumented immigrants and residents not enrolled in social security (43, 44). However, it is important to avoid any similar measures that exclude migrants in the future as these infectious conditions do not discriminate on race, religion, or immigration status, accordingly, all members of the community need adequate access to health care to limit the spread of infections. Unlike the financial crisis of 2008, the current crisis is based on a public health emergency, which cannot be achieved without considering the health of migrant populations. Accordingly, any potential economic crisis should not allow for reforms that may negatively affect the health of migrants. Additionally, migrant and ethnic minority workers are highly represented in key sectors that were crucial during the lockdown, such as the healthcare sector, the food industry as well as agriculture. This means that migrant workers may face another type of inequality, in which they are at higher risk of occupational exposure to the infection during employment. Migrants workers account for 13% of all key workers in the EU, and this percentage can reach up to more than 30% in specific key sectors such as cleaners and domestic helpers (45). In the UK, BAME who represent only 21% of the National Health Service members (46), account for 61% of mortalities from COVID-19 (47). Likewise, the previously described Somali community in Nordic countries, which is disproportionately affected by COVID-19, also tend to work in similar sectors with high risk of exposure to COVID-19 infection (28).

Moreover, lack of access to healthcare and economic instability during the COVID-19 epidemic shows how mental health among migrants could be affected due to concerns of contracting the disease, income loss, or mental stress imposed by the lockdown. In particular, migrants and refugees may exhibit symptoms of mental disorders due to exposure to unpleasant events before, during or after migration (48). Accordingly, mental stress due to the current COVID-19 pandemic may add to previously existing symptoms.

We should bear in mind that if we aim to achieve the control of SARS-CoV-2 infection, no population at high risk of infection can be excluded. Although no single solution can address these deeply rooted health and socioeconomic inequalities among migrants in Europe, urgent initiatives should be adopted to mitigate the effect of COVID-19 among these vulnerable populations. These initiatives should emphasize the importance of interventions that guarantee the full integration of migrants and ethnic minorities in the health system regardless of their migration status. Policies should also be developed to ensure access to information and prevention services without bearing any financial or legal consequences, especially among undocumented migrants. The assessment of migrants' health needs and barriers to access health services is critical for mitigating any future public health crisis such as COVID-19. Protective plans should be introduced to prevent the pandemic's long-term side effects on different vital aspects of life among migrants, such as unemployment, poor housing, and mental health issues. Moreover, governments are required to provide more comprehensive strategies and solutions to the apparent pre-existing health socioeconomic inequalities among migrants in Europe. The development of any strategies or policies against COVID-19 that do not consider an equitable health coverage of the whole population, including migrants, will be doomed to failure and will halt any efforts in controlling the spread of the infection.

## Author Contributions

AS conceived the work, reviewed the literature and wrote the manuscript. BP and MM supervised the work and wrote the manuscript. All authors have agreed on the final version of the manuscript.

## Conflict of Interest

The authors declare that the research was conducted in the absence of any commercial or financial relationships that could be construed as a potential conflict of interest.
